# Effects of Erythropoietin on Gliogenesis during Cerebral Ischemic/Reperfusion Recovery in Adult Mice

**DOI:** 10.14336/AD.2016.1209

**Published:** 2017-07-21

**Authors:** Rongliang Wang, Jincheng Li, Yunxia Duan, Zhen Tao, Haiping Zhao, Yumin Luo

**Affiliations:** ^1^Cerebrovascular Diseases Research Institute and Department of Neurology, Xuanwu Hospital of Capital Medical University, Beijing 100053, China; ^2^Beijing Institute for Brain Disorders, Beijing 100053, China; ^3^Beijing Key Laboratory of Translational Medicine for Cerebrovascular Diseases, Beijing 100053, China; ^4^Department of Neurology, Zibo Central Hospital, Zibo 255036, China

**Keywords:** Brain ischemia, Erythropoietin, Microglial polarization, Gliogenesis, White matter injury

## Abstract

Erythropoietin (EPO) promotes oligodendrogenesis and attenuates white matter injury in neonatal rats. However, it is unknown whether this effect extends to adult mice and whether EPO regulate microglia polarization after ischemic stroke. Male adult C57BL/6 mice (25–30g) were subjected to 45 min of middle cerebral artery occlusion (MCAO). EPO (5000 IU/kg) or saline was injected intraperitoneally every other day after reperfusion. Neurological function was evaluated using the rotarod test at 1, 3, 7 and 14 days after MCAO. Brain tissue loss volume was determined by hematoxylin-eosin staining. Immunofluorescence staining and Western blot were also used to assess the severity of white matter injury and phenotypic changes in microglia/macrophages. Bromodeoxyuridine (BrdU) was injected intraperitoneally daily for 1 week to analyze the number of newly proliferating glia cells (oligodendrocytes, microglia, and astrocytes). We found that EPO significantly reduced Brain tissue loss volume, ameliorated white matter injury, and improved neurobehavioral outcomes at 14 days after MCAO (*P*<0.05). In addition, EPO also increased the number of newly generated oligodendrocytes and attenuated the rapid hypertrophy and hyperplasia of microglia and astrocytes after ischemic stroke (*P*<0.05). Furthermore, EPO reduced M1 microglia and increased M2 microglia (*P*<0.05). Taken together, our results suggest that EPO treatment improves white matter integrity after cerebral ischemia, which could be attributed to EPO attenuating gliosis and facilitating the microglial polarization toward the beneficial M2 phenotype to promote oligodendrogenesis.

Cerebral ischemia results not only in gray matter damage, but also in severe white matter injury, thereby disrupting signal transmission and eliciting poor functional outcomes. At present, most preclinical cerebral ischemia studies greatly emphasize gray matter over white matter, which may contribute to the disappointing results in clinical trials over the years [1]. Previous studies have indicated that white matter injury nearly accounts for half of the infarct volume [2] and recent studies highlighted the importance of white matter integrity in recovery after stroke [3]. The predominant cell type in the cerebral white matter consists of glia such as oligodendrocytes and astrocytes, which together provide the myelin for neuronal fibers and maintain a cellular and extracellular environment necessary for neuronal functioning.

Cerebral ischemic stroke can induce the proliferation of oligodendrocyte precursor cells (OPCs) [4], but these newly generated OPCs fail to develop into mature oligodendrocytes, thereby resulting in insufficient remyelination and white matter repair [5, 6]. An increasing number of studies agree that microglia can assume diverse phenotypes and engage different functions in response to specific microenvironment [7]. The dual roles of polarized microglia have already been reported in stroke, brain trauma and cerebral hemorrhage [8-10]. Notably, recent studies indicated that the M2 microglia phenotype could promote oligodendrocyte regeneration, which was otherwise impaired by the M1 phenotype [9, 11]. Those findings strongly suggest that microglial polarization helps determine oligodendrocyte fate and white matter integrity after brain injuries [12]. Thus, preventing the M2 to M1 shift with microglia-related therapies may present an attractive opportunity to benefit not only victims of cerebral ischemic injury, but also other CNS disorders. Erythropoietin has emerged as a promising candidate for neuroprotection in animal models of ischemia and in stroke patients for years. Delayed administration of EPO stimulates oligodendrogenesis and attenuates white matter injury after hypoxic/ischemic in neonatal rats [13]. Moreover, previously studies also indicated that EPO modulates neuroinflammation by acting directly on glia [14]. However, the effects of EPO on microglial polarization, oligodendrogenesis and white matter integrity after ischemic stroke in adult mice are currently unknown.

In this study, we used a well-established mouse model of transient focal cerebral ischemia to analyze the effects of EPO on gliogenesis during white matter repair and microglia polarization after brain injury. Our results show that EPO treatment not only decreases gliosis, but also enhances the generation of new oligodendrocytes and remyelination process, therefore promoting white matter repair at 14 days following middle cerebral artery occlusion (MCAO) in the adult mouse brain. We further demonstrate that EPO-induced microglial polarization toward the M2 phenotype could contribute to enhancing oligodendrocyte differentiation.

## MATERIALS AND METHODS

### Animal grouping and middle cerebral artery occlusion

Young male C57BL/6 mice (2-month-old) were purchased from Vital River Laboratory Animal Technology Co. Ltd. All experimental protocols were approved by the Institutional Animal Care and Use Committee of Capital Medical University. Focal cerebral ischemia was induced by transient middle cerebral artery (MCA) occlusion. Briefly, mice were anaesthetized with enflurane and the right common carotid artery (CCA) was exposed. A nylon filament with a 0.19 mm diameter silicon tip was inserted to obstruct the flow of blood to the right MCA for a period of 45 min after which it was removed to allow for reperfusion. The local cerebral blood flow was observed using a transcranial laser Doppler (LDF, PeriFlux System 5000; Perimed, Sweden) to confirm occlusion of the MCA. Body temperature was monitored with a rectal probe and maintained at 37.0 ± 0.5°C using a heating lamp during surgery. Mean arterial blood pressure was monitored during MCAO through a tail cuff, and arterial blood gases were analyzed at 15 min after the onset of ischemia.

Twenty-seven male mice were randomly divided into 3 groups: (1) sham group (N=9): the mice underwent the same procedure as other groups without insertion of the suture; (2) ischemic/reperfusion (I/R) + vehicle group (N=9; I/R+Veh): after MCAO, mice were administered saline through intraperitoneal injection at the beginning of reperfusion; (3) I/R + EPO group (N=9; I/R+EPO): after MCAO, mice were treated with EPO (5000 IU/kg) through intraperitoneal injection at the beginning of reperfusion.

### BrdU injection

To trace the generation of newly proliferating cells, mice intraperitoneally injected with the thymidine analog, 5′-bromo-2′-deoxy-uridine (BrdU, 50 mg/kg), daily for 7 days beginning at 1 day after MCAO.

### Accelerating rotarod test

Motor coordination was assessed using an automated rotating rod. Mice were placed on an accelerating rotating rod (4 to 40 rpm over 120 s) and their latency to fall off the rod was recorded. Preoperative training was performed for 3 days with 3 daily trials. Postoperative testing was performed at 1, 3, 7, and 14 days after MCAO, 3 trials per day, with the mean latency to fall analyzed thereafter.

### Hematoxylin-Eosin (HE) staining

All mice were transcardially perfused with 4% paraformaldehyde (PFA) in phosphate-buffered saline (PBS) and their brains removed. Brains were post-fixed in 4% PFA for 24 hrs, and then immersed in 30% sucrose solution in 4% PFA for 48 hrs. After post-fixation and cryoprotection, the brain sections were cut coronally to a thickness of 15-µm using a cryostat vibratome (Ultapro 5000, USA). Brain tissue loss volume was determined by calculating the amount of viable tissue using H&E staining. Briefly, the sections were stained with H&E, then dehydrated through graded concentrations of ethanol followed by xylene. Finally, the sections were mounted with a coverslip and air dried overnight. Images were captured by a blinded investigator with a microscope. A series of 6 sections in around the MCA territory were selected in each mouse brain.

The brain tissue loss area for each section was measured using the following equation: (H&E-positive staining in contralateral hemisphere - H&E-positive staining in ipsilateral hemisphere)/H&E-positive staining in contralateral hemisphere × 100%. Mean Brain tissue loss volume was then determined by multiplying the mean loss area by the thickness of the evaluated tissue.

### Immunofluorescence staining and quantification

Immunofluorescent staining was performed as previously described [15]. The frozen sections were blocked with 0.3% (w/v) BSA in PBS at room temperature for 1 hr. To evaluate white matter injury, the sections were incubated with primary antibodies against neurofilament 200 (NF-200; mouse; 1:200; Abcam) and myelin basic protein (MBP; rabbit; 1:200; Abcam) overnight at 4°C. After incubating with fluorescent-conjugated secondary IgG antibodies, including FITC (1:400; Jackson Immunoresearch) and Cy3 (1:400; Jackson Immunoresearch), all sections were counterstained with 4′,6-diamidino-2-phenylindole (DAPI). The images were digitized using an Olympus Fluoview FV1000 microscope (Olympus, Japan) with FV10-ASW 2.0 software. The mean intensity value of NF-200 and MBP staining was calculated in the region of interest (ROI), which included the cortex (CTX), corpus callosum (CC) and striatum (ST) then analyzed with ImageJ software as previously reported [13]. White matter damage was expressed as the relative ratio of NF-200 to MBP staining.

For visualization of newborn cells, sections were pretreated with 2 N HCl followed by 0.1 mol/L boric acid (pH 8.5) then incubated with anti-BrdU (1:200; BD Biosciences). Gliogenesis was determined by the co-immunostaining for BrdU and mature oligodendrocytes (CNPase, 1:200; Novus Biologicals), microglia (Iba1, 1:200, Wako Pure Chemical Industries), and astrocytes (GFAP, 1:200; Santa Cruz Biotechnology). Three randomly selected microscopic fields in the peri-infract area were analyzed by a blinded investigator. Immunopositive cell counts were presented as the mean number of cells per square millimeter.

**Figure 1.**
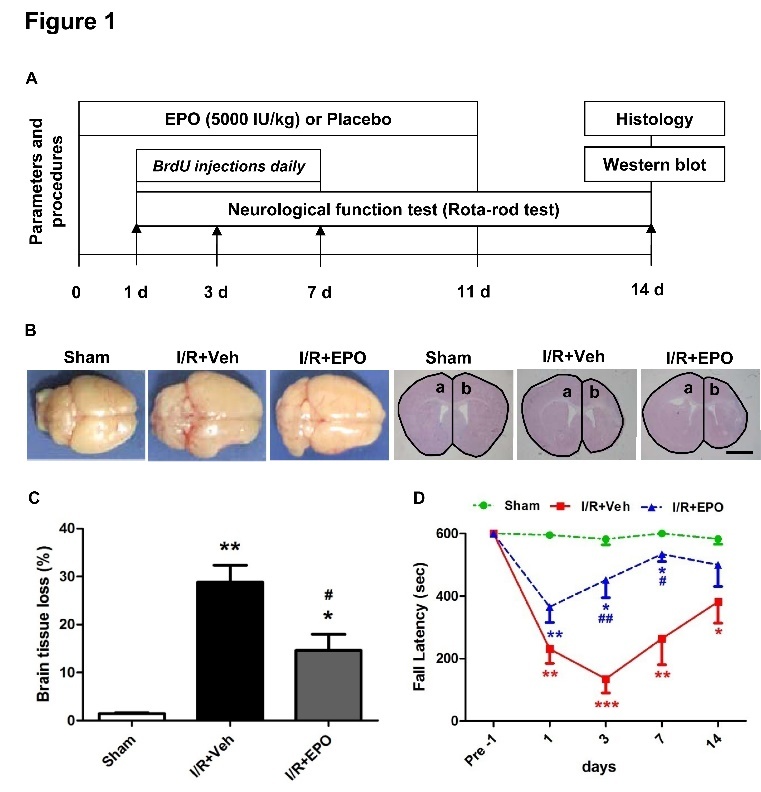
EPO reduces brain tissue loss volume and improves recovery of neurological function after MCAO Experimental design. Mice received EPO or saline intraperitoneally (i.p.) every other day until day 11 (**A**). Representative pictures and quantification of brain tissue loss volume at 14 days after I/R. (**B, C**). The brain tissue loss for each section was measured using this equation: mean brain tissue loss volume (%) = (Area a - Area b)/Area a × 100%. Scale bar, 1 mm. n=4 per group. Neurological function was assessed by the Rotarod test (**D**). *P≤0.05, **P≤0.01 *vs.* sham; ^#^*P*≤0.05 *vs.* I/R vehicle. n=9 per group.

### Western blot analysis

The ipsilateral brain tissue was homogenized in lysis buffer (RIPA) containing protease inhibitors (Thermo, USA). After sonication, protein concentrations were calculated and SDS-PAGE carried out. The nitrocellulose membranes were incubated with the following primary antibodies at 4 °C overnight: anti-MBP (1:1000, Abcam, USA), anti-CNPase (1:1000, Novus, USA), anti-CD16 (1:1000, Abcam, USA), anti-CD11b (1:1000, Abcam, USA), anti-CD206 (1:1000, Abcam, USA), and β-actin (1:1000, Santa Cruz, USA). Following incubation with appropriate horseradish peroxidase (HRP)-conjugated secondary antibodies (1:5000, Abgent, USA), the immunoblots were visualized with a computerized image analysis system (Fluro Chen 2.0, USA). The integrated density values were calculated with the software AlphaEaseFC and normalized to β-actin.

### Statistical analysis

All data are presented as mean ± standard error of the mean (SEM). Data with two groups were analyzed with the *Student’s t-test* (non-directional). For differences between multiple groups, results were analyzed with one-way or two-way analysis of variance (ANOVA), followed by Tukey’s post hoc test. Differences were considered significant at *P*≤0.05.

**Figure 2.**
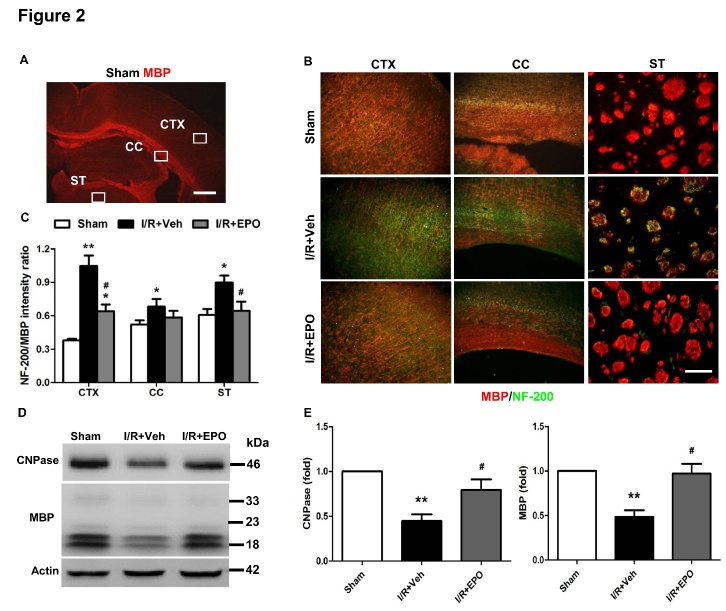
EPO attenuates white matter injury after MCAO MBP (red) and NF-200 (green) immunostaining 14 days after I/R (**A-C**). Boxes in (**A**) indicate the selected areas of the cortex (CTX), corpus callosum (CC), and striatum (ST). Scale bar, 1 mm. Immunostaining of MBP and NF-200 in the CTX, CC, and ST in the ipsilateral hemisphere (**B**). Scale bar, 50 μm. Quantification of the relative ratio of NF-200 *vs.* MBP immunostaining intensity in ipsilateral hemispheres (**C**). Relative expression levels of myelin proteins (CNPase and MBP) in the ipsilateral brain tissue of different groups (**D**). β-actin served as a loading control. n=4 per group. Representative western blots for results in D (**E-F**). *P≤0.05, **P≤0.01 *vs.* sham; ^#^*P*≤0.05 *vs.* I/R vehicle. n=4 per group.

## RESULTS

### EPO improves recovery of neurological function and reduces brain tissue loss after I/R injury

Multiple injections of EPO (5000 IU/kg) and BrdU (50 mg/kg) were administered intraperitoneally as diagrammed (Fig. 1A). Neurological function was assessed using the rotarod test. We found that EPO treatment significantly reduced motor deficits after ischemic stroke as demonstrated by an increased latency to fall off the rotarod at 3 and 7 days post-injury (both *P*<0.05; Fig. 1D). We then evaluated brain tissue loss volume by H&E staining. Corresponding with the neurological function results, EPO treatment significantly reduced brain tissue loss volume compared with vehicle-treated mice at 14 days after MCAO (*P*<0.05; Fig. 1B and C).

### EPO promotes white matter integrity after I/R injury

We examined damage to the axons and myelin sheath in the cortex (CTX), corpus callosum (CC) and striatum (ST) (Fig. 2 A) by assessing the number of MBP-positive cells, a marker of myelination, and NF-200-positive cells, a marker of axons. At 14 days after cerebral ischemic injury, NF-200 immunoreactivity was abundant within the CTX and ST, reflecting the demyelination of axons EPO significantly reduced the NF-200/MBP ratio (*P*<0.05; Fig. 2B and C), suggesting long-term preservation (or remyelination) of myelinated axons. Compared to the vehicle group, the myelin-associated proteins (MBP and CNPase) were markedly increased at day 14 following EPO treatment (*P*<0.05; Fig. 2D, E).

**Figure 3.**
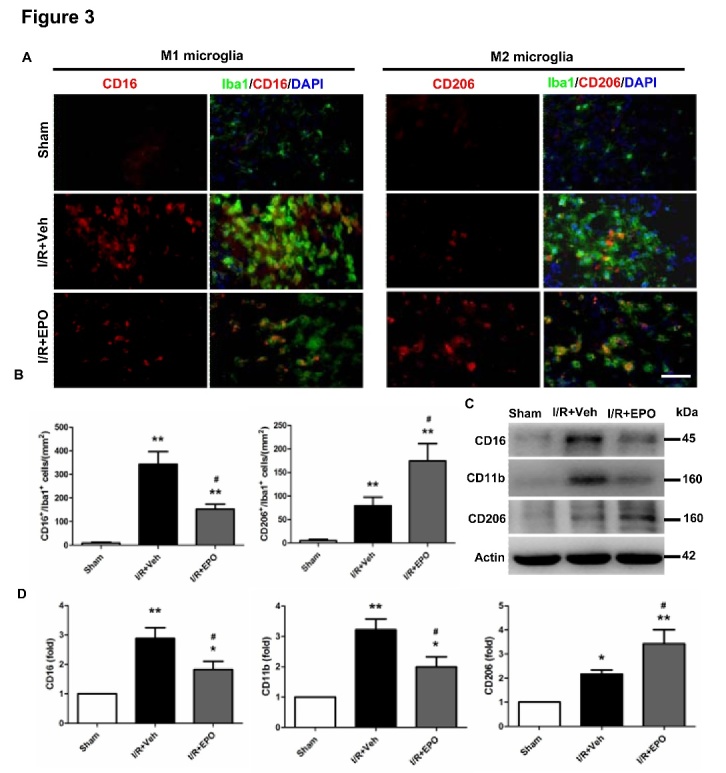
EPO promotes the M2 microglial phenotype after MCAO Double immunofluorescent staining for M1 marker (CD16) or M2 marker (CD206) (red) with Iba1 marker (green) for activated microglia in the peri-infarct region at 14 days following I/R injury (**A**). Scale bar, 100 μm. Quantification of microglia cells immunopositive for CD16 and CD206 (**B**). Protein expression levels of M1 (CD16 and CD11b) and M2 (CD206) markers at 14 days following I/R injury (**C, D**). *P≤0.05, **P≤0.01 *vs.* sham; #*P*≤0.05 *vs.* I/R vehicle. n=4 per group.

### EPO drives M2 microglia polarization after I/R injury

To specifically evaluate the polarization states of microglia/macrophage after cerebral ischemic injury in different groups, we further examined the expression of M1 (CD16) and M2 (CD206) markers in Iba1^+^ microglia by double immunofluorescent staining and Western blotting (Fig. 3). The Iba1 immunostaining revealed that EPO treatment attenuated microglial activation and thereby hypertrophy around the peri-infract areas at 14 days after MCAO (Fig. 3A). The number of CD16-positive microglia in the I/R+Veh group was much higher than in sham-operated mice (*P*<0.05; Fig. 3A and B). Notably, EPO treatment saw the decrease in the number of M1 microglia (CD16^+^/Iba1^+^ cells) but an increase in the number of M2 microglia (CD206^+^/Iba1^+^ cells) (*P*<0.05; Fig. 3A and B). To verify the immunofluorescent staining results, we measured the expression of microglial markers by Western blot. The results indicated that after EPO treatment, the protein expression levels of the M1 markers CD16 and CD11b significantly decreased, while the M2 marker, CD206, markedly increased in the ipsilateral cortex at day 14 after cerebral ischemic injury (*P*<0.05; Fig. 3C and D). Taken together, these findings suggest that EPO promotes microglial polarization to the M2 phenotype 14 days after ischemia.

**Figure 4.**
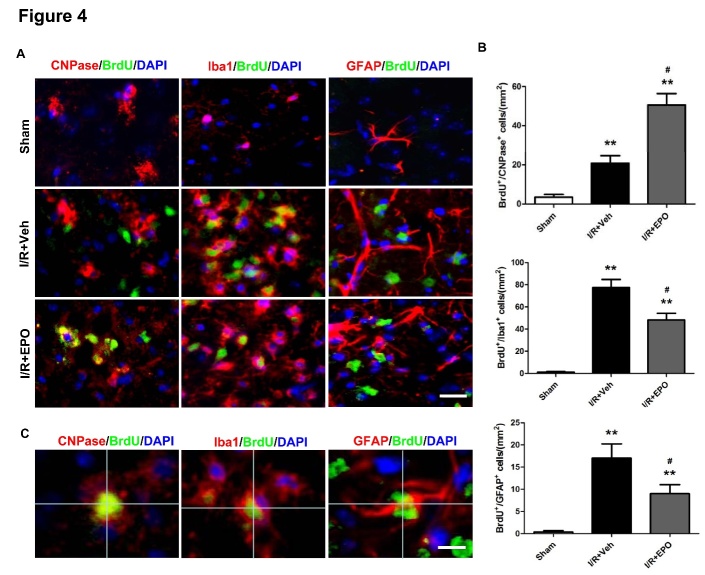
EPO enhances the generation of new oligodendrocytes and decreases gliogenesis after MCAO Colocalization of 5′-bromo-deoxyuridine (BrdU; green) and glia cells (CNPase, Iba1 and GFAP; red) in the peri-infarct region at 14 d following I/R injury (**A**). Scale bar, 100 μm. Representative image showing colocalization of BrdU^+^/CNPase^+^, BrdU^+^/Iba1^+^, BrdU^+^/GFAP^+^, and DAPI (blue) staining at high magnification (**B**). Scale bar, 20 μm. Numbers of BrdU and glia double-positive cells expressed as cells/mm^2^ (**C**). *P≤0.05, **P≤0.01 *vs.* sham; ^#^*P*≤0.05 *vs.* I/R vehicle. n=4 per group.

### EPO enhances oligodendrogenesis and decreases gliogenesis after MCAO

Regeneration of mature myelinating oligodendrocytes is essential for remyelination and functional recovery after cerebral ischemia. Thus, we sought to determine whether EPO treatment can influence oligodendrogenesis after MCAO, thereby facilitating white matter restoration. Brain sections were double-stained with BrdU and anti-CNPase, a marker for mature oligodendrocyte cell body. Increase in the number of co-localized CNPase and BrdU positive cells were detected in peri-infarct areas in vehicle-treated MCAO mice compared with sham controls (*P*<0.05; Fig. 4A), suggesting that spontaneous generation of new oligodendrocytes occurs at 14 days after MCAO. Treatment with EPO further augmented the number of new mature oligodendrocytes, as evidenced by the increase in BrdU^+^/CNPase^+^ cells in the peri-infarct areas when compared with the I/R+Veh group (*P*<0.05; Fig. 4A and B).

To determine the effects of EPO on gliogenesis, the sections were also double-stained with BrdU and Iba1 (a marker of mature microglia) or GFAP (a marker of mature astrocytes). In contrast, the number of the BrdU^+^/Iba1^+^ cells in I/R+EPO groups were reduced significantly compared with the I/R+Veh group (*P*<0.05; Fig. 4A and B). The decreasing trends in the number of BrdU^+^/GFAP^+^ cells of all groups were consistent with that of BrdU^+^/Iba1^+^ cells observed (*P*<0.05; Fig. 4A and B). In addition, enhancement of GFAP immunoreactivity was seen in both gray and white matter of MCAO mice14 days post-injury. After EPO-treatment, astrocytes appeared sparser and thinner compared with I/R mice (Fig. 4A). Interestingly, the numbers of the BrdU^+^/GFAP^+^ cells are much less than BrdU^+^/Iba1^+^ cells in all groups (Fig. 4A).

## DISCUSSION

The current study demonstrated the neuroprotective effects of EPO associated with its ability to attenuate I/R-induced rapid hypertrophy and hyperplasia of microglia and astrocytes as well as the facilitation of microglial polarization toward the M2 phenotype, which promotes mature myelinating oligodendrocyte regeneration to result in white matter reparation and the observed improvement in neurological functional outcomes 14 days after cerebral ischemia in adult mice.

White matter contains exclusively axons and their glial cell counterparts including oligodendrocytes, microglia, and astrocytes [16]. It is highly vulnerable to ischemic injury in neonates and adults alike, and contributes to the deterioration of functional outcomes. Many experimental studies have confirmed the efficacy of EPO in neonatal hypoxic-ischemic (H/I) brain injury in rodents [13], including improvements in long-term neurobehavioral outcomes. One mechanism of the beneficial effects exerted by EPO in the neonatal brain is the enhancement of oligodendrogenesis and recovery of injured white matter. A previous study suggested that delayed EPO treatment significantly increased the ratio of MBP to NF-200 staining in the CTX and CC 14 days after neonatal hypoxic-ischemic injury [13]. The neuroprotective effects of EPO in adult mice seen in our current study wherein EPO significantly reduced the NF-200/MBP ratio in the ST and CTX at 14 days after MCAO. Moreover, the expression levels of myelin-associated proteins (MBP and CNPase) are markedly increased in the EPO-treated group, which suggests long-term preservation of myelinated axons in adult mice. Interestingly, previous findings suggested that the adult brain has a limited capacity for white matter repair; the compensatory increases in MBP is insufficient for restoring white matter integrity at day 14 after cerebral ischemia [9]. Multiple factors may cause regenerative failure in the adult CNS, including the weakness of intrinsic growth capacities and the inhibitory extrinsic environment [17]. Objectively speaking, data from our current study does not clearly explain the differential results we observed in our adult mouse model *vs.* the neonatal mouse after cerebral ischemia reperfusion injury. One possible explanation may be directly related to the common pathological changes triggered by ischemic stroke, such as the proliferation of activated microglia and astrogliosis [16]. Moreover, astrocytes produce extracellular hyaluronic acid both in both neonatal and adult ischemia [18], which plays a role in glial scar formation and impairs the OPC regenerative response. In fact, studies have shown that EPO can alleviate these pathological changes. Further study is therefore warranted to better understand the EPO’s differential effects in both the neonatal and adult ischemic/hypoxia mouse models.

Inflammation is a normal physiologic response to injury and is necessary for tissue healing. However, when neuroinflammation is severe or chronic, it can produce deleterious effects, including impairing neurogenesis [19] and preventing axon regeneration [20]. Microglia has similar characteristics with systemic macrophages, such as playing an important role in neuroinflammation and converting into the M1 and M2 reactive states [12]. Studies have shown that there is a mixture of both M1- and M2-activated microglia at sites of injury. M1 microglia increase secretion of pro-inflammatory mediators, which impair axon regrowth [21]. In contrast, M2 microglia can mediate neuroprotective functions and promote neurogenesis [10, 19]. In a traumatic brain injury model, the cortical lesion led to chronic and persistent M1-primed activation that lasted for months to years [20]; this chronic inflammation increases white matter injury and reduces self-restorative abilities [9]. Recent studies have shown that the microglial phenotypic switch to M2 encourages oligodendrocyte differentiation, modulates white matter repair and axonal remyelination [11, 22, 23]. However, the M2-like activation tends to switch to M1-like activation within one week to result in more deleterious effects, and has already been reported in models of stroke, trauma, hemorrhage and spinal cord injury [8-10, 21]. Thus, preventing the M2 to M1 transition and maintaining the M2 microglia phenotype may be an attractive opportunity to benefit brain injury. Consistent with previous findings [24], our data suggest that EPO maintains microglia in a normal state and prevents microglial proliferation. However, as far as we know, there is no report on whether EPO could modulate the switches in microglial polarization. In the current study, we demonstrate a shift in the M1 to M2 phenotypes after EPO treatment at the infarct border after brain injury. The number of M2 microglia was elevated, while the number of M1 microglia returned to near pre-injury levels at 14 days following cerebral ischemia.

Oligodendrocytes influence neuronal conductance by forming insulating myelin sheaths, which is critical for OPC differentiation and oligodendrogenesis. Generation of new re-myelinating oligodendrocytes is essential for white matter repair after ischemic injury. Studies have shown that EPO stimulates angiogenesis and neurogenesis after cerebral injury, especially in the neonatal rodent [24]. Moreover, EPO can induce the proliferation of oligodendrocyte precursor cells (OPCs) after cerebral hypoxic/ischemic injury [13]. Although detection of the precursor cells by specific markers such as BrdU and CNPase can lend some support for oligodendrogenesis, it is not strong evidence for the generation of new oligodendrocytes because many precursor cells die or never become fully mature and functional cells [25]. In our study, we confirm the renewal of glia cells based on mature cell markers (Iba1, CNPase and GFAP). As expected, an increasing number of newborn oligodendrocytes around the infarct areas in the ipsilateral CTX and the restoration of myelin in the ipsilateral ST and CTX were observed in the EPO-treated group. The result demonstrates that EPO enhances oligodendrogenesis to restore white matter integrity at 14 days after MCAO.

Astrocytes, microglia and oligodendrocytes are the main glial cells in the CNS and they influence neural plasticity and repair in different ways [26]. In a variety of CNS injuries, astrocytes have been reported respond to tissue damage by undergoing rapid hypertrophy and hyperplasia [27]. The reactive astrocytes secrete molecules, such as chondroitin sulfate proteoglycans (CSPGs) that not only inhibits axonal regeneration at the site of the glial scar, but also influence several neuronal and glial physiological properties through the modification of the extracellular microenvironment [28]. In our study, we identified an increased generation of astrocytes following MCAO, consistent with astrogliosis and the glial scar. Previous studies have shown that the acute administration of recombinant human erythropoietin (rhEPO) in a rat model of spinal cord injury (SCI) reduces lesion size, attenuates gliosis and microglia/macrophage activation as well as enhance myelinogenesis and improve locomotor outcome [29]. In line with this finding, our results show that EPO treatment not only attenuates astrogliosis, but also decreases generation of new microglia and astrocytes in the brain parenchyma at 14 days following cerebral ischemia. The reduction of the extent of astrogliosis after EPO treatment likely contributed to the oligodendrogenesis. The increased number of BrdU^+^ cells in the peri-infarct area indicated that EPO treatment either stimulated proliferation or DNA repair capacity after cerebral ischemia. Interestingly, we found a significant difference in BrdU labeling responses where the number of the BrdU^+^/GFAP^+^ cells are much less than BrdU^+^/Iba1^+^ cells in different groups. Our result is different from other reports [30,31], but is partly consistent with a previous study, which demonstrated that EPO increased the number of BrdU^+^/NeuN^+^ double-labeled cells in the ischemic hemisphere 14 days after stroke, whereas there were only a few BrdU^+^/GFAP^+^ positive cells, suggesting that EPO promoted progenitor differentiation towards a neuronal lineage [32]. The differential actions of EPO on microglia and astrocyte generation after ischemic injury may be the result of varying degrees of cerebral injury as well as different time points of BrdU injection and observation. Thus, the underlying mechanism will need to be explored more in-depth in the future.

The present study demonstrates for the first time that EPO prevents the M2 to M1 shift and maintains the M2 microglia phenotype. Meanwhile, EPO increases oligodendrogenesis and decreases gliosis to contribute to white matter repair 14 days after stroke in adult mice. This study provides a novel insight into EPO treatment for ischemic stroke and explores EPO’s effects on microglial polarization.
